# Investigation of the Dynamic Behaviour of H_2_ and D_2_ in a Kinetic Quantum Sieving System

**DOI:** 10.1021/acsami.3c17965

**Published:** 2024-02-29

**Authors:** Dankun Yang, Sebastien Rochat, Matthew Krzystyniak, Alexander Kulak, Jacques Olivier, Valeska P. Ting, Mi Tian

**Affiliations:** † Department of Mechanical Engineering, University of Bristol, Bristol BS8 1TR, U.K.; ‡ School of Engineering Mathematics and Technology, University of Bristol, Bristol BS8 1TW, U.K.; § School of Chemistry, University of Bristol, Bristol BS8 1TS, U.K.; ∥ ISIS Facility, Rutherford Appleton Laboratory, Didcot OX11 0QX, U.K.; ⊥ School of Chemistry, University of Leeds, Leeds LS2 9JT, U.K.; #.Institute Laue Langevin, GrenobleF-38042, France; ∇.School of Engineering, Computing and Cybernetics & Research School of Chemistry, Australian National University, Canberra 0200, Australia; ○.Department of Engineering, University of Exeter, ExeterEX4 4QF, U.K.

**Keywords:** porous organic cage, quantum sieving, hydrogen isotope separation, quasielastic neutron scattering, kinetic quantum sieving, nuclear quantum effects, kinetic analysis

## Abstract

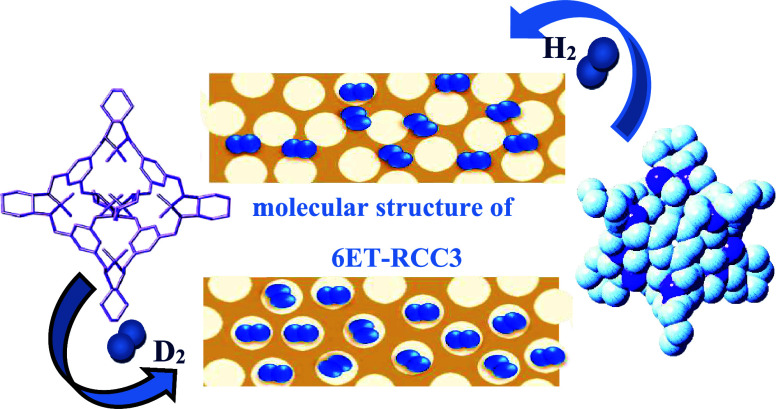

Porous organic cages (POCs) are nanoporous materials composed of discrete molecular units that have uniformly distributed functional pores. The intrinsic porosity of these structures can be tuned accurately at the nanoscale by altering the size of the porous molecules, particularly to an optimal size of 3.6 Å, to harness the kinetic quantum sieving effect. Previous research on POCs for isotope separation has predominantly centered on differences in the quantities of adsorbed isotopes. However, nuclear quantum effects also contribute significantly to the dynamics of the sorption process, offering additional opportunities for separating H_2_ and D_2_ at practical operational temperatures. In this study, our investigations into H_2_ and D_2_ sorption on POC samples revealed a higher uptake of D_2_ compared to that of H_2_ under identical conditions. We employed quasi-elastic neutron scattering to study the diffusion processes of D_2_ and H_2_ in the POCs across various temperature and pressure ranges. Additionally, neutron Compton scattering was utilized to measure the values of the nuclear zero-point energy of individual isotopic species in D_2_ and H_2._ The results indicate that the diffusion coefficient of D_2_ is approximately one-sixth that of H_2_ in the POC due to the nuclear quantum effect. Furthermore, the results reveal that at 77 K, D_2_ has longer residence times compared to H_2_ when moving from pore to pore. Consequently, using the kinetic difference of H_2_ and D_2_ in a porous POC system enables hydrogen isotope separation using a temperature or pressure swing system at around liquid nitrogen temperatures.

## Introduction

Demand for hydrogen and its isotopes is predicted to surge in the coming decades due to the increasing demand for green energies. In addition to protium (H), a widely used green vector, deuterium (D) and tritium (T) are predicted to play important roles in the energy supply as well. The lower incoherent cross section of deuterium (D), as compared to that of protium, makes it valuable as a moderator in many nuclear systems,^1^ while tritium (T) functions as the input fuel in nuclear fusion reactors and is a highly important feedstock for future low-carbon fusion energy.^2^ However, separating the hydrogen isotopes is challenging, as the isotopes share very similar physicochemical characteristics. As a result, energy-intensive processes such as cryogenic distillation and Girdler-sulfide processes are currently used for hydrogen isotope separation.^3,4^ Transitioning from these traditional approaches, current research is exploring the use of quantum behaviors to facilitate isotope separation, moving away from solely relying on nearly identical physiochemical properties.

Confined nanospaces offer special options for hydrogen isotope separation by creating a quantum system and enabling separation based on the difference in nuclear zero-point energy^5^ (ZPE) or in the adsorption enthalpies due to chemical affinity between metal sites of frameworks and the gas molecules^6^ under cryogenic conditions. The separation of hydrogen isotopes in a nanoporous system using the nuclear quantum effect generated from different ZPEs or adsorption enthalpies is known as quantum sieving. Different porous materials can achieve several types of sieving appropriate for isotope separation, such as kinetic quantum sieving^7^ (KQS) and chemical affinity quantum sieving^8^ (CAQS). Therefore, some microporous materials that can enable quantum sieving have become competitive candidates in hydrogen isotope separation. A lot of candidates^9^ showed good potential for separating hydrogen isotopes by quantitative differences in the adsorption processes, such as zeolites,^10−12^ covalent organic frameworks^13^ (COFs), metal–organic frameworks^14^ (MOFs), and porous organic cages^15,16^ (POCs). Challenges of the CAQS encompass a range of factors. First, suitable materials for CAQS are selective, limiting the diversity of applicable materials. Overlaps in chemical affinities for different isotopes can reduce the separation efficiency. Moreover, CAQS’s performance is sensitive to temperature fluctuations, requiring stringent temperature control.

**Figure 1:**
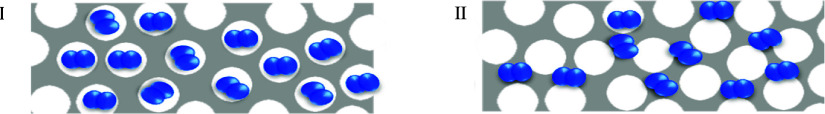
Two different types of diffusion can exist in a porous quantum system: (I) gas molecules (blue spheres) fully adsorbed within the pores. (II) Gas molecules diffuse through interstitial spaces due to the quantum effect.

However, the kinetic difference from the nuclear quantum effect can be underestimated, as most of the research focuses on the differences in the quantity of each of the isotopes adsorbed or desorbed in a given material under set conditions. The existence of quantum barriers can change the diffusion pathways of the gas molecules. Instead of being adsorbed on the porous surface, some gas molecules can diffuse through interstitial spaces between lattices (Figure 1). To better understand the kinetic behavior of the isotopes in the porous systems, we focus on the dynamic differences between H_2_ and D_2_ in the sorption process and the kinetic behavior of adsorbed H_2_ and D_2_ in the porous quantum system.

When the pore width is comparable to the de Broglie wavelength of the isotopes in porous materials, a quantum effect (barrier)^17^ can emerge, especially at cryogenic temperatures.^18^ Therefore, in this study, we selected the POC 6ET-RCC3^19^ (chemical formula C_84_H_120_N_12_) to explore the kinetic behavior of D_2_ and H_2_ under KQS conditions. The particular porous material offers a narrow pore-size distribution centered around 3.6–4.4 Å in diameter, which maximizes the probability of generating quantum effects.^20^

In scenarios where gases like H_2_ and D_2_ interact with materials or are confined, particularly within adsorbent pores, their behaviors can be significantly influenced by quantum effects. This results in the lighter isotope molecules diffusing more swiftly inside porous materials at lower temperatures than their heavier counterparts. Theoretically, the thermal energy required to overcome the quantum barrier for H_2_ should be much higher than for D_2_, and H_2_ should perform differently compared to D_2_.^5^ Such behavior leads to kinetic quantum isotope molecular sieving. Given its lighter mass, H_2_ is notably more susceptible to quantum effects than D_2_. This could indicate that H_2_ molecules, influenced by these quantum mechanics principles, navigate between different sites in the sample in ways that contradict conventional wisdom.

However, the amount of the isotope effect on the nuclear ZPE can depend on factors other than the isotope mass. A subtle interplay between the local curve of the potential energy surface (PES) experienced by a given isotopic species, the degree of spatial and chemical confinement, the temperature, and the isotope mass determines the net value of the nuclear ZPE.^21^ This nontrivial behavior can be monitored by a unique technique of neutron Compton scattering (NCS), which measures the amounts of isotopic ZPEs and curvatures of local PES they experience in an isotope-resolved manner.^22,23^

This study aimed to explore the kinetics and diffusion processes of H_2_ and D_2_ in a confined nanoporous quantum system. The dynamic behaviors of H_2_ and D_2_ during the sorption process in the porous quantum system were studied by comparing fractional uptakes^24^ generated from kinetic analysis and the calculation of ZPEs from measured values of the widths of nuclear momentum distributions (NMDs)^25^ through NCS. The potential effect of the quantum barrier was probed by analyzing the kinetic behavior of H_2_ and D_2_ using quasielastic neutron scattering^26^ (QENS) by comparing elastic incoherent structure factor^27^ (EISF) and calculating self-diffusion parameters^28^ to elucidate which mechanisms the different isotopes are using.

## Experimental Methods

### Synthesis of the 6ET-RCC3

Porous organic cage 6ET-RCC3 was synthesized following the method described in Liu et al.^20^ (See Supporting Information for details).

### Powder X-ray Diffraction (PXRD), Scanning Electron Microscopy (SEM), and Nuclear Magnetic Resonance (NMR)

PXRD^29^ was used to test the stability and retention of crystallinity before and after different experiments using a Bruker D8 Advance X-ray diffractometer in flat plate geometry with Cu Kα source wavelength λ = 1.5418 Å at 293 K over the range 5–50° and 2θ with a step size of 0.02 2θ.

SEM^30^ images were acquired at the University of Leeds using a field emission SEM FEI Nova 450. Samples were dispersed by using ethanol onto a silicon wafer and attached to a stub. Images were prepared after coating with iridium (4 nm) at 3 kV.

^1^H NMR analyses were performed with a Bruker 400 spectrometer. Samples were dissolved in deuterated chloroform and tested at room temperature by using residual protonated solvent as a reference. Results were analyzed using MestReNova.^31^

### CO_2_ and N_2_ Low-Pressure Gas Sorption for Pore Size Distribution

CO_2_ and N_2_ sorption were measured on a Micromeritics 3-Flex volumetric gas sorption analyzer from 0 to 0.9 bar at 273 and 77 K, respectively, to generate the pore size distribution based on nonlocal density function theory (NLDFT)^32,33^ and the BET^34^ and Langmuir^35^ surface areas using the 3-Flex software from Micromeritics. The sample was degassed at 80 °C for over 10 h at a high vacuum of 10^–9^ bar before testing to remove the moisture without damaging the organic structure.

### H_2_ and D_2_ Gas Sorption for Dynamic and Isothermal Analysis

The kinetics of uptake was calculated based on H_2_ and D_2_ sorption experiments performed on a Micromeritics 3-Flex volumetric gas sorption analyzer from 0 to 1 bar at 77 K. The POC samples were completely degassed in situ at 80 °C for over 10 h before gas adsorption measurement.

Equation 1 was used to fit the adsorbent fractional uptake^36^ (θ_t_) of each step:
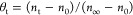
1where *n*0, *n*, and *n*_∞_ are the adsorbed volume at the beginning of the adsorption step, during equilibration, and at equilibrium, respectively.

The fractional uptake can also be calculated using the linear driving force (LDF)^37^ model (eq 2)
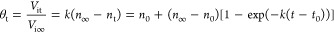
2where *V*_it_ is the uptake volume of the gas at time *t*, *t*_0_ is the time in the beginning pressure step, *n*_t_ is the adsorbed volume at time *t*; *V*_i∞_ is the uptake volume at the equilibrium of the measured pressure step, and *n*_∞_ is the absorbed amount at equilibrium. Therefore, under isothermal conditions for pure gas input, *V*_it_/*V*_i∞_ is also known as the adsorbent fractional uptake (θ_t_) for the measured pressure, and *k* can be regarded as the kinetic rate constant.

The LDF model, which is based on Fick’s Law and describes the dynamic behavior of diffusion in a matrix structure, is a multiplied form of the general diffusion equation (Fickian Diffusion)^38^ but is suitable for analyzing isothermal adsorption of the pure guest molecules in a single porous adsorbent.^39^ Furthermore, the fractional uptake, which represents the mass change in each step, is also known as the external mass transfer coefficient^37^ (*k*) of H_2_ and D_2_ in the pores. The mass transfer coefficient, *k*, is linearly related to the diffusivity of the adsorbed gases,^37^ indicating the adsorption rate and the affinity of the cage for gases under specific conditions.

Both H_2_ and D_2_ used in the sorption experiments were supplied by Air Liquide with a purity of 99.999%. Before measurement, the samples were degassed under high vacuum (10^–7^ mbar) at 80 °C for over 12 h to ensure that they were fully degassed without damaging the organic structure (as confirmed by PXRD).

### Neutron Compton Scattering

To obtain information about the widths of NMDs and mean kinetic energieswhichyield the values of the nuclear ZPEs of the individual isotopic species in the adsorbed guest molecules within the microporous host, we employed neutron Compton scattering (NCS).^40,41^ The NCS data were collected on the Vesuvio spectrometer operating at the ISIS Neutron and Muon Source, UK. Around 1 g of previously degassed material was placed in an aluminum holder, resulting in a 0.5 mm-thick sample with a cross-sectional area of 40 cm^2^, a value sufficient to cover the entire circular beam. The Vesuvio experiments were performed at 30, 50, and 77 K under 0.9 bar of gas (H_2_ or D_2_). For each experiment, the value of the integrated proton current within the ISIS synchrotron of ca. 3000 μAh. In all cases, each subsequent hour of beam time (8 h) corresponded to an additional value of 180 μAh of the integrated proton current. The data recorded in both front and backscattering configurations when the H_2_/D_2_ mixture was present were obtained by subtracting the data recorded for samples containing the gas in a container from those of the empty container. In the fitting process, the technique of stoichiometric constraint was applied^42,43^ for the integral intensities of the recoil peaks of all atomic species with the exception of aluminum. The value of the Al nuclear momentum width was fixed at 14 ± 1 Å^–1^, which was obtained from a series of calibration experiments.^44^ Data treatment routines implemented in the MantidPlot computational environment were employed.^43−45^

### Quasi-Elastic Neutron Scattering

Quasielastic neutron scattering was measured by the IN5 instrument at the Institute Laue-Langevin, Grenoble, France, with an incident source wavelength of 6 Å. The IN5 offers an energy resolution of 85 μeV fwhm with a momentum transfer range from 0.2 to 2 Å^–1^ with a step size of 0.1 Å^–1^. Before attaching the sample to the central capillary, 0.375 g of 6ET-RCC3 was degassed by heating at 353 K in a vacuum oven for 18 h to remove moisture and adsorbed gases. During the QENS measurement, an HTP-1 system manufactured by Hiden Isochema was used to load the gases in the 6ET-RCC3 with hydrogen isotope pressures of 0.1, 0.5, and 1 bar at 50 and 77 K. The sample contribution and experimental distortion were corrected by measuring the sample in the absence of any gases at 50 and 77 K.

Vanadium was measured at 298 K as the experimental resolution fitted by the resolution function, the Delta function. After deducting the sample’s contribution from the raw data, the QENS spectra represented only adsorbed gas molecules. Lorentzian^25,44,45^ peak was applied to represent the diffusion that happens during the adsorption process, and the Gaussian^47−50^ equation, which represents the diffusion among the interstitial spaces of the lattices, was applied to model the movement of the molecules prevented from being adsorbed due to the quantum barrier. The data was then analyzed using DAVE to calculate the fitted half-width at half-maximum (HWHM) and elastic incoherent structure factor (EISF)^51,52^ (eq 3).
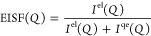
3where *I*^el^(*Q*) and *I*^qe^(*Q*) are the elastic and quasi-elastic intensities at specific *Q* values. Therefore, EISF at a particular *Q* value can be calculated by the ratio of the area of the elastic peak to the sum of all incoherent peaks (eq 4),^53^ where *A*_E_ is the area under the elastic peak and *A*_Q_ is the area under quasielastic peaks. By calculation of the ratio of the elastic intensity to the total intensity, the EISF can be plotted for different *Q* values.
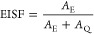
4

Also, to simulate the two different types of movement of H_2_, two different jumping or diffusion models needed to be applied, while for D_2_, which only behaved as pure adsorption, one model was applied to the results.

## Results

### Characterization of the Porous Organic Cage (6ET-RCC3)

**Figure 2:**
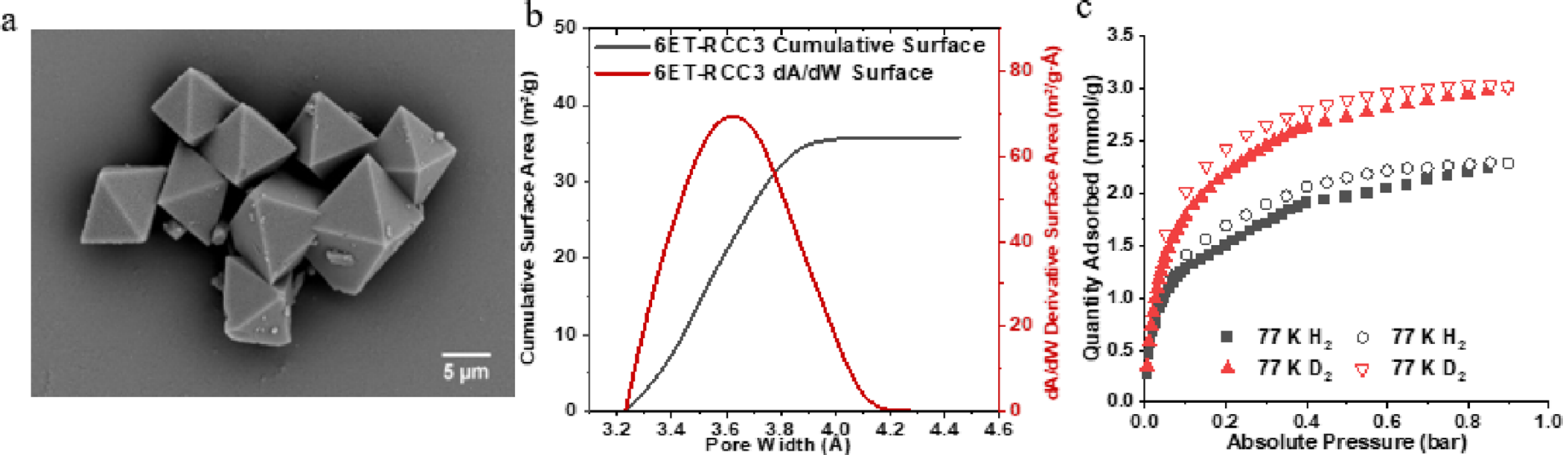
(a) SEM image of 6ET-RCC3 crystals, (b) pore size distribution and the derivative pore volume of 6ET-RCC3 calculated by NLDFT from 273 K CO_2_ sorption, and (c) H_2_ isotherm (black) and D_2_ isotherm at 77 K of the 6ET-RCC3, closed sympols for adsorption, and open sympols for desorption.

The synthesized organic cage 6ET-RCC3 was analyzed by ^1^H NMR (Figure S1), and its spectrum was found to be in good agreement with the prior literature.^54^ The expected crystalline pattern and hexahedral crystal structure were confirmed by powder X-ray diffraction (Figure S2) and SEM, respectively (Figure 2a). Characterization with NMR and PXRD was repeated postmeasurement (i.e., after gas uptake and QENS) to confirm the stability of the cage material under the experimental conditions.

### Pore Size Distribution and Surface Area

Nitrogen adsorption experiments at 77 K suggested the BET surface area was around 23.11 m^2^/g, but the measurement was known to be of limited use to probe micropores with diameters below 5 Å due to slow kinetics. Therefore, the nanopore distribution was measured by CO_2_ adsorption experiments at 273 K with a Langmuir surface around 85.13 m^2^/g. The pore size distribution of 6ET-RCC3 is illustrated in Figure 2b, indicating a concentration of pore sizes primarily around 3.6 Å. This specific pore size in 6ET-RCC3 is significant as it meets the fundamental requirements for quantum sieving. The concept of kinetic quantum sieving (KQS) in nanoporous solids, first introduced by Liu et al.^20^ becomes significantly effective when the confining space is comparable to the molecular hydrogen’s thermal de Broglie wavelength.^55^ For optimal KQS, ultrafine pore apertures, approximately 3 Å, are essential.^9,20,56^ Therefore, the pore size of approximately 3.6 Å in 6ET-RCC3 is significant, suggesting that 6ET-RCC3 is a suitable candidate for KQS-related applications.

### Low-Pressure H_2_ and D_2_ Isotherms

**Figure 3:**
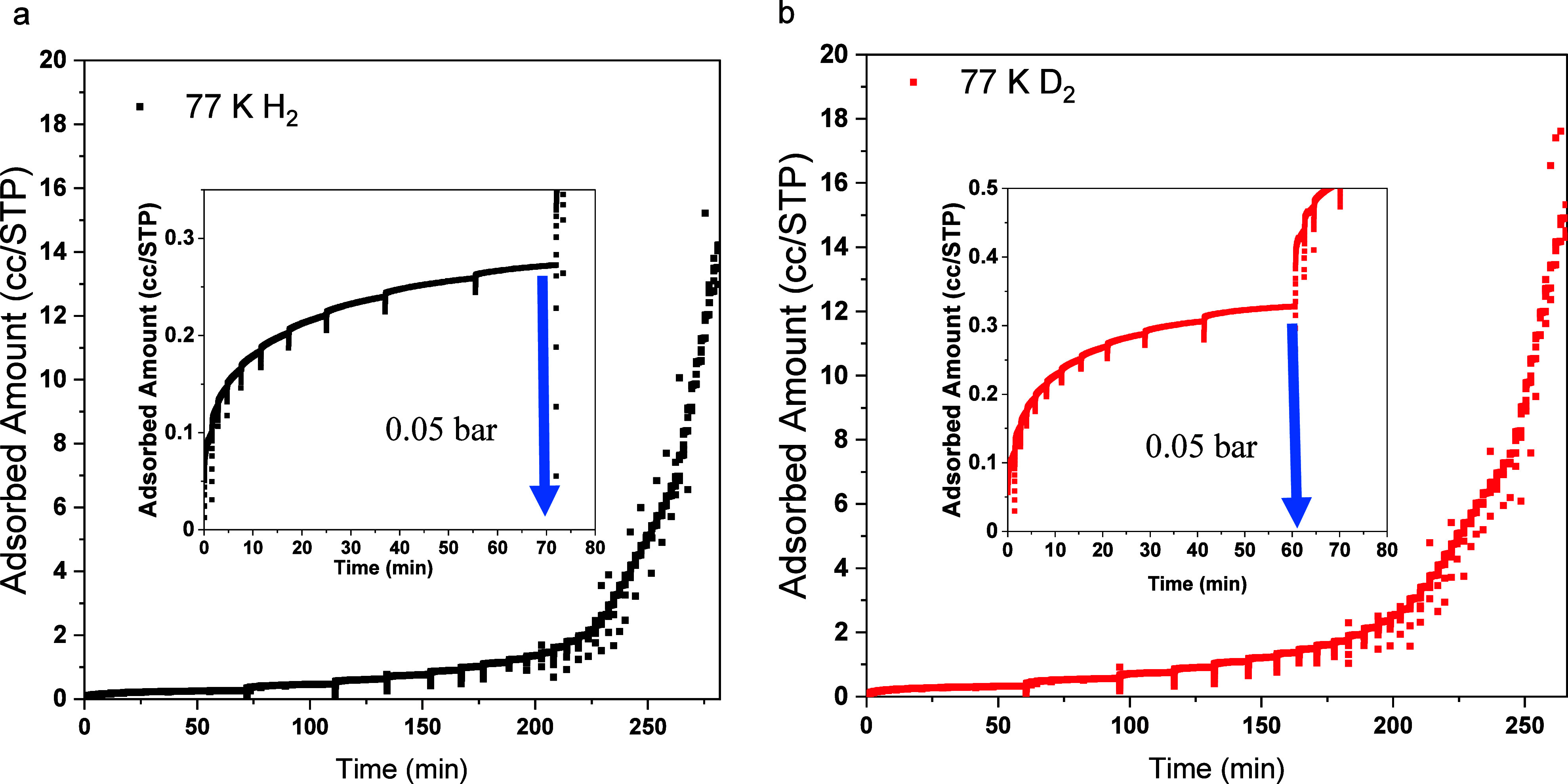
H2 and D2 gas uptake on the POC as a function of time at 77 K up to 1 bar: (a) H2; (b) D2.

The pure H_2_ and D_2_ gas adsorption isotherms on 6ET-RCC3 were individually measured at 77 K up to 0.9 bar, as shown in Figure 2c. Given the characteristic of small-pored materials like 6ET-RCC3 requiring extended periods to attain adsorption equilibrium, the gas adsorption experiments were designed to ensure the recording of data once 99% equilibration was achieved without premature termination, as shown in Figure 3. Consequently, the isotherms reflect the gases’ adsorption at this near-complete equilibration point. Both H_2_ and D_2_ isotherms demonstrate complete reversibility, suggesting that the process is physical sorption driven by the van der Waals force. This claim is further substantiated by the reproducibility of H_2_ adsorption under identical conditions (Figure S1b) and the consistency in the PXRD patterns before and after the gas adsorption process (Figure S2).

Significantly, the data indicate a notable difference in adsorption capacity between the two gases at 77 K and 0.9 bar: the material adsorbs D_2_ at 3 mmol/g, which is 33% higher than its adsorption of H_2_. This notable discrepancy, despite the molecular sizes of H_2_ and D_2_ being very similar at liquid nitrogen temperature, highlights the material’s affinity for adsorbing D_2_. The higher adsorption capacity for D_2_ underscores the potential of 6ET-RCC3 in selectively facilitating D_2_ separation from H_2_ at 77 K. To further understand the mechanism behind this selective adsorption and explore the kinetics of H_2_ and D_2_ interaction with 6ET-RCC3, additional investigative studies were undertaken.

### Kinetics H_2_ and D_2_ Sorption

Herein, we designed experiments to explore the kinetic difference between H_2_ and D_2_ to define more practical separation conditions for the porous systems by analyzing the kinetics of each adsorption step (Figure S3). D_2_ reached equilibrium within a notably shorter duration of 250 min, compared to the 280 min required for H_2_ at 77 K, as shown in Figure 3. This indicates that under the same conditions D_2_ attains equilibrium faster than H_2_. A notable distinction is observed in the low-pressure range (<0.05 bar), where D_2_ reached a pressure of 0.05 bar in under 60 min, whereas H_2_ exceeded 70 min to attain the same level. This behavior indicates a kinetic preference for D_2_ on the adsorbent’s porous surfaces during the adsorption process. The mass transfer coefficients of the gas molecules, detailed in Table 1, further substantiate this kinetic favorability.

**Table 1:** External Mass Transfer Coefficient (*k*) Calculated by the LDF Model of H_2_ and D_2_ at 77 K

step pressure at 77 K (bar)	*k* of H_2_ (s^–1^)	*k* of D_2_ (s^–1^)
0.05	0.41 ± 0.02	0.58 ± 0.03
0.1	1.85 ± 0.13	1.96 ± 0.11
0.5	1.95 ± 0.05	1.97 ± 0.06
0.9	2.32 ± 0.22	2.37 ± 0.17

Combining the information generated from the adsorption isotherm, it appears that the quantity difference of H_2_ and D_2_ at 77 K benefitted most at 0.9 bar, while the kinetic difference is more observable at the low-pressure range when the porous surface is more available to the gas molecules. To have a deeper understanding of how H_2_ and D_2_ behave kinetically in the porous system, neutron experiments under these conditions are performed.

## Neutron Compton Scattering

The magnitude of the ZPE isotope effect measured in the neutron Compton experiment can be used for the system under investigation as a tool for model selection for the shape of the underlying potential energy surface locally experienced by H and D in the H_2_ and D_2_ molecules undergoing radial motion in the pores. Values of the standard deviations of NMDs for H and D at *T* = 30, 50, and 77K, denoted as σ, along with the nuclear ZPEs, as derived from the analysis of the NCS data, are presented in Table 2 and Figure S4. To ensure the gas molecules probed by the experiments were fully adsorbed by the porous system, the experiments were performed with 0.9 bar of H_2_ and D_2_, respectively, with the measurement temperatures increasing from the starting point of 30 K (scattering curve shown in Figures S5 and S6). As our experiments rely on physisorption only, in the presence of a pure gas environment (H_2_ or D_2_), the differences between the values of the ZPEs and the NMD widths are expected to correlate with differences in the behavior of H_2_ and D_2_ during the sorption process.^40^

**Table 2:** Values of the NMD Widths and Nuclear ZPEs of H and D at 0.9 bar and 30, 50, and 77 K Obtained from the NCS Experiments

*T* (K)	σ_H_ (Å^–1^)	σ_D_ (Å^–1^)	σ_D_/σ_H_	ZPE of H (meV)	ZPE of D (meV)
30	4.2 ± 0.2	6.3 ± 1.7	1.5 ± 0.3	218.5 ± 20.8	245.9 ± 72.0
50	4.3 ± 0.3	5.2 ± 1.4	1.2 ± 0.3	229.0 ± 32.0	167.5 ± 90.0
77	4.0 ± 0.3	7.2 ± 2.3	1.8 ± 0.3	198.2 ± 29.6	321.2 ± 102.0

The value of the ratio of the NMD width of D to that of H differs from the theoretical value for the harmonic potential value of = 1.1892 and, in consequence, the ratio of the vibrational zero-point energies, ZPE_H_/ZPE_D_, differs from the value of = 1.41. For quantum confining potentials with increasingly steep walls, the value of ZPE_H_/ZPE_D_ increases from  and reaches the limiting value of 2, in the case of the square-well potential^8^ or in the case of the cylindrical square-well potential.^57^ The interplay between the temperature effect and the van der Waals type of interaction between the molecular species present in the pores and the pore walls produces local effective confining potentials experienced by H_2_ and D_2_ in their highly quantized radial motion that change their degree of anharmonicity as the temperature changes. This leads to concomitant changes in the magnitude of the isotope effect for the vibrational ZPE that are different from the idealized case of the cylindrical square wall potential. The values of the ZPEs of H are greater than their counterparts for D at *T* = 50 K, proving that the effective height of the barrier for diffusion of H is reduced at the intermediate temperature, while at *T* = 30 K, H and D exhibit similar effective barrier heights. That indicates the probability of generating quantum effects for H at 50 K, or even 77 K.

The momentum of the atoms further confirmed the potential for generating KQS in the POC system. By virtue of the Heisenberg uncertainty principle, the product of the standard deviations of the position and momentum distributions in the ground state of the quantum harmonic oscillator is equal to *ℏ*/2 (in the units used in the theory of the NCS,^46^). Thus, the momentum distribution of H_2_ at 77 K (σ_H_ approximately 4.0 Å^–1^) corresponds to the proton position distribution of 0.25 Å for every H in the H_2_ molecule. For D in the D_2_ molecule at 77 K, the width of the momentum distribution is 7.2 Å^–1^, and the position distribution of D in D_2_ has a width of 0.139 Å. This renders the effective size of the H_2_ molecule larger than that of the D_2_ molecule, and the molecular size commensurate with effective pore size satisfied the conditions of generating KQS in the porous system at 77 K.

Furthermore, the momentum (σ) and ZPE of D decreased when the temperature was reduced from 77 to 50 K, adhering to the macroscopic diffusion rules. However, when the temperature was further reduced to 30 K, the NMD width and ZPE started to increase, suggesting the formation of the quantum barrier for D might have started at below 50 K. These findings emphasize the potential of harnessing quantum-induced kinetic variations for hydrogen isotope separation at 77 K.

### Quasi-Elastic Neutron Scattering

QENS was employed due to its unique capacity for identifying kinetic differences between adsorbed hydrogen isotopes in porous quantum systems. The experiments enabled the detailed probing of rotational and diffusive motions of the adsorbed gas molecules in the pores, helping to identify H_2_ or D_2_ molecules undergoing different types of diffusion in the kinetic quantum sieving system.

**Figure 4:**
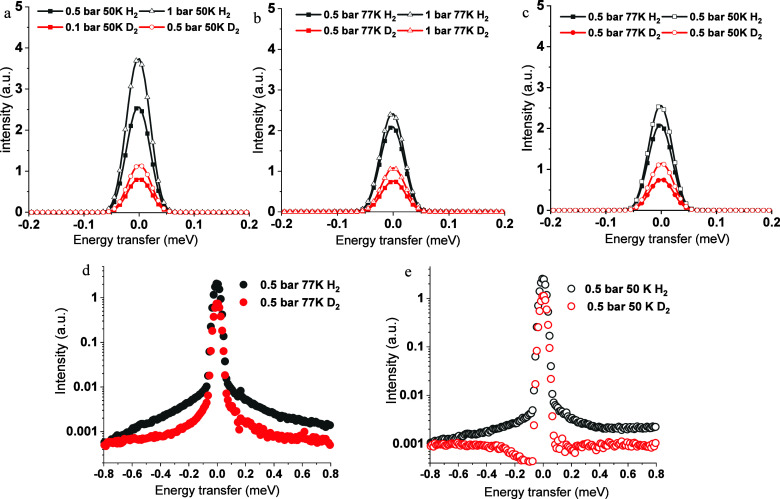
QENS spectra of H_2_ (black) and D_2_ (red) at different temperatures and pressures: (a) 0.5 bar (solid),1 bar (open) H_2_ and 0.1 bar (solid), 0.5 bar (open) D_2_ spectra 50 K. (b) 0.5 bar (solid) and 1 bar (open) H_2_ and D_2_ spectra at 77 K. (c) H_2_ and D_2_ spectra at 0.5 bar 77 K (solid), 50 K (open). (d) H_2_ and D_2_ spectra at 0.5 bar 77 K in log intensity scale. (e) H_2_ and D_2_ spectra at 0.5 bar 50 K in log intensity scale.

Broadening of the QENS spectra is observed at all Q values for both H_2_ and D_2_ adsorbed within the POC system at 50 and 77 K, as shown in Figure 4. Under the consistent measurement conditions, the H_2_ spectra exhibited greater intensity compared to D_2_, accompanied by a broader width of the peaks in Figure 4a,b. This phenomenon is attributed to the high incoherent cross section of H (80.27 barns), which is approximately 40 times greater than that of D.^58^ Reducing temperature from 77 to 50 K led to augmented intensity, evident in Figure 4c, correlating with increased H_2_ and D_2_ gas uptake. Similarly, an increase in pressure included a similar increase in peak intensity as the amounts of adsorption increased with pressure. As depicted in Figure 4d,e, there is the evident broadening of both H_2_ and D_2_ spectra at 0.5 bar for both 77 and 50 K.

Remarkably, the full-width half-maximum (fwhm) generated from the quasielastic peak of hydrogen at both 77 and 50 K is more pronounced than that of D_2_, indicating a more rapid molecular motion of H_2_ within the samples compared to D_2_ (Figure 4). Even at a comparably elevated temperature of 77 K, distinctions in the dynamics of H_2_ and D_2_ remain apparent. This underscores the distinctive behaviors of the adsorbed hydrogen isotopes in the porous system.

**Figure 5:**
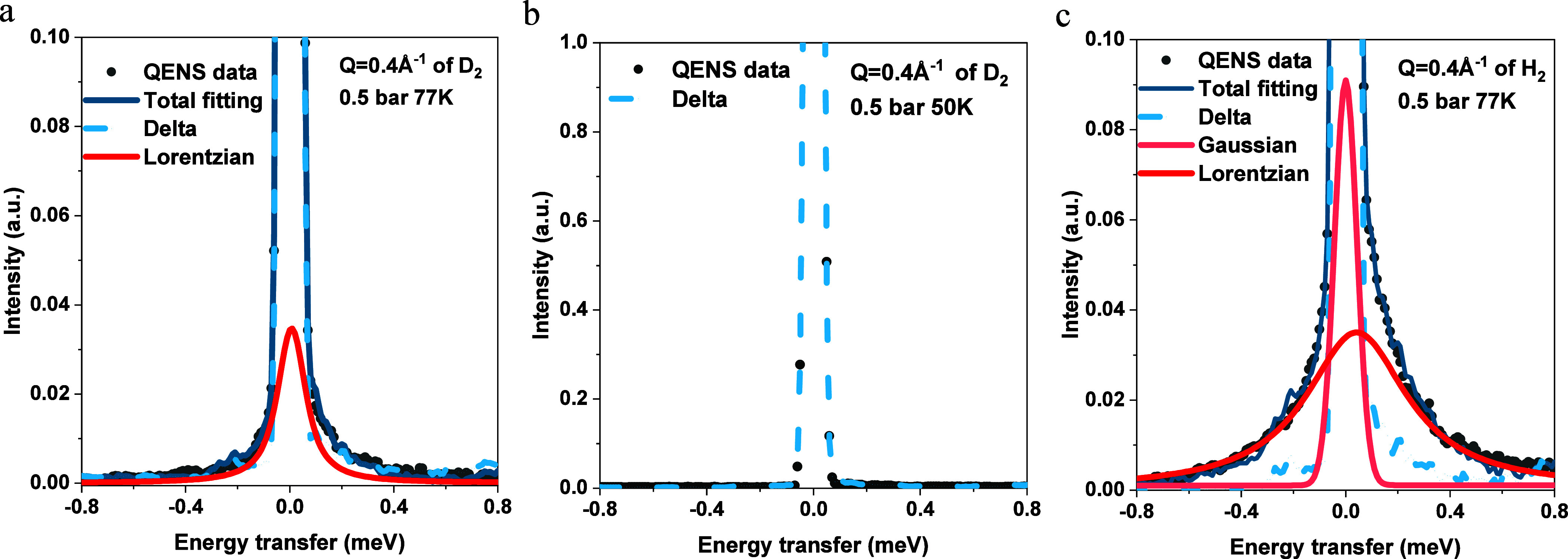
Deconvoluted 0.5 bar spectra of H_2_ and D_2_: (a) Fitted D_2_ spectrum fitted with the delta function (dot blue) and Lorentzian (red) at *Q* = 0.4 Å^–1^ 77 K. (b) Fitted D_2_ spectrum with delta (dot blue) at *Q* = 0.4 Å^–1^ 50 K. (c) Deconvoluted H_2_ spectrum fitted with delta (dot blue), Gaussian (dot light red), and Lorentzian (red) at *Q* = 0.4 Å^–1^ 77 K.

Since QENS data are collected once adsorption has reached equilibrium, it offers insights into the diffusion coefficient. This coefficient describes the likelihood and rate at which gas molecules move within the material after equilibrium has been established. It is a direct measure of the mobility of gas molecules within the porous matrix under equilibrium conditions. This information can be acquired by fitting the quasielastic peaks of the spectra (detailed in Figures S6 and S7). In the fitting process, the experimentally measured resolution function was convoluted with a delta function to represent elastic scattering. The Lorentzian equation was employed to characterize the self-rotational movement of adsorbed molecules, signifying a relatively static movement confined within pores. For D_2_, both ZPE and momentum distributions (Table 2) suggested it undergoing the traditional sorption process at both 50 and 77 K. Building on this, the QENS spectra for D_2_ were consistently and aptly modeled using only the Lorentzian curve (Figure 5a).

This suggests a more localized D_2_, particularly when confined to a conclusion that aligns with previous research findings.^20^ It is worth noting that as illustrated in Figure 5b, the QENS spectrum of D_2_ at 0.5 bar and 50 K can be exclusively represented by a delta fit (Figure 5b). This suggests that there is no observable broadening of D_2_ when confined in pores at 50 K, indicating D_2_ was highly localized in pores (Figure S7 and S11) without generating the quantum effect.

However, H_2_ might already experience the quantum effect at 77K based on the NCS results with increased ZPE and momentum distribution widths, so an additional equation is required to represent the molecules that diffuse among the interstitial spaces rather than the confined pores. Thus, after exploring a wide array of fitting models, we include a Gaussian equation to represent the jump-diffusion behavior affected by the quantum effect, as depicted in Figure 5c.

**Figure 6:**
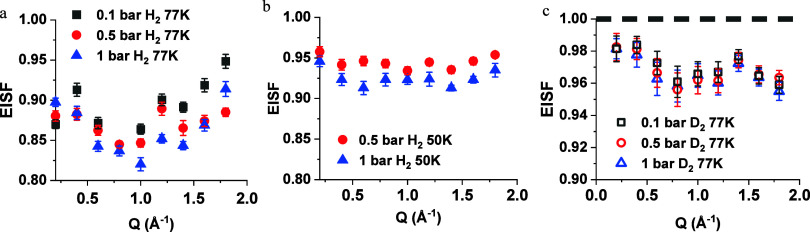
Elastic incoherent structure factor (EISF) of (a) H_2_ (solid) at 50 K 0.5 bar (red) and 1 bar (blue); (b) H_2_ (solid) at 50 K 1 bar (black), 0.5 bar (red), 0.1 bar (black); (c) D_2_ (open) at 0.1 bar (black), 0.5 bar (red) and 1 bar (blue) at 77K and the dashed line for D_2_ at 50 K.

As seen from the collected QENS spectra under the same conditions, the HWHM (half width of half-maximum) of quasi-broadening generated from adsorbed H_2_ was wider than that of D_2_, again proving that the movement of H_2_ in the POC is much more rapid at the same temperature. This has also been proven by the elastic incoherent structure factor (EISF), which is subsequently employed to ascertain the overall fraction of the elastic phase for both H_2_ and D_2_, as well as their respective diffusion geometries, as shown in Figure 6.

**Figure 7:**
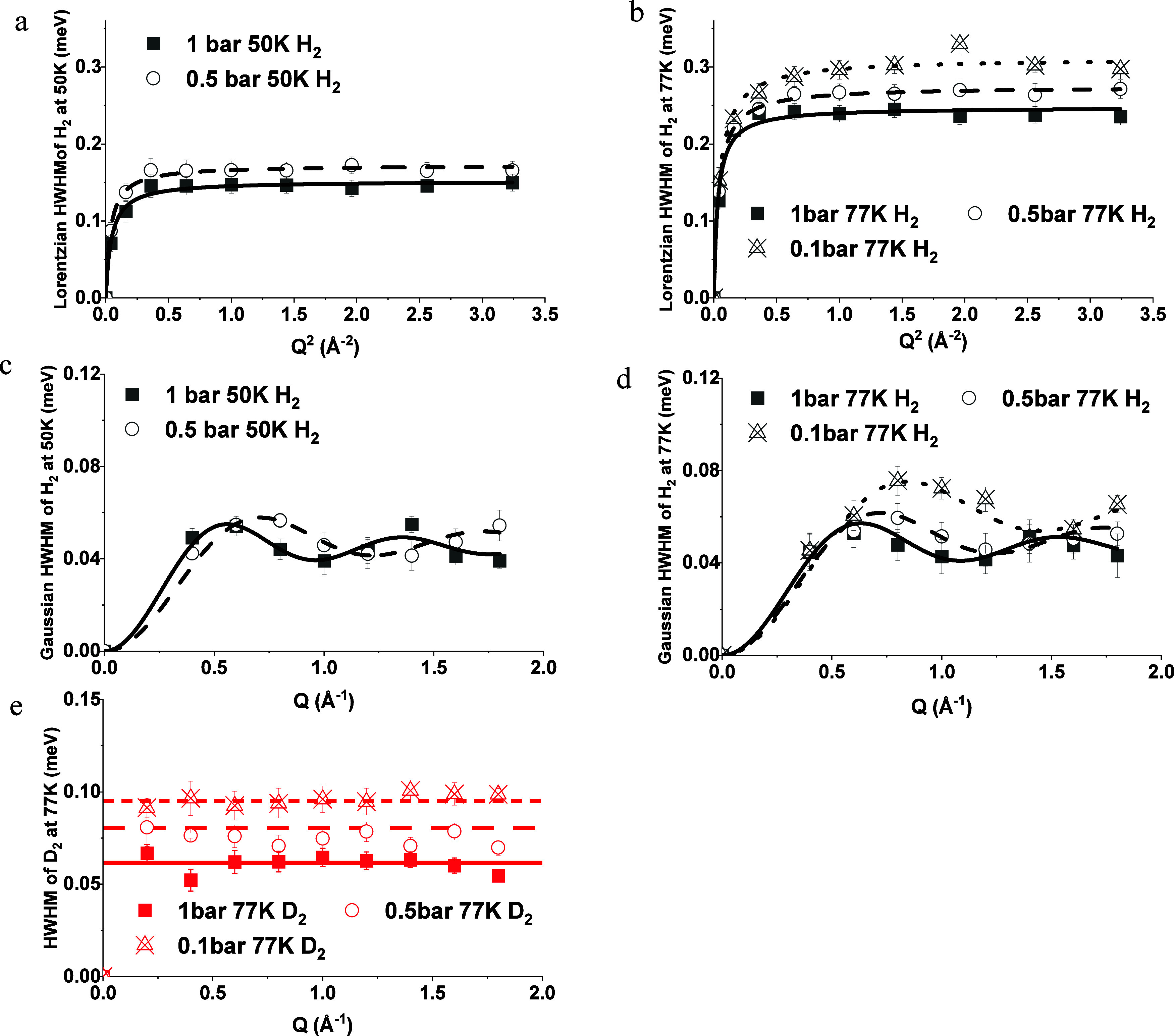
*Q*-Dependence of the HWHM broadening of the Lorentzian components of QENS spectra of (a) H_2_ at 50 and (b) at 77 K at 1 (solid square), 0.5 (open square), and 0.1 bar (cross triangle) with the Singwi–Sjölander model; *Q*-dependence of the HWHM broadening of Gaussian components of H_2_ fitted with the Chudley–Elliot model at (c) 50 K and at 1 (solid square), 0.5 (open circle), and 0.1 bar (cross triangle), (d) at 77 K and 1 (solid square) and 0.5 bar (open circle). (e) HWHM broadening of the Lorentzian components of QENS spectra of D_2_ at 77 K and at 0.1 (cross triangle), 0.5 (open square), and 1 bar (solid square).

Notably, as pressure and temperature increase, the EISF of H_2_ decreases (visualized in Figure 6a,b). This decrease could be attributed to a shift in localized motion caused by the increase in pressure and temperature. On the other hand, the EISF of D_2_ remains notably consistent irrespective of the pressure changes, as illustrated in Figure 6c. This consistency suggests a uniform confinement mechanism for D_2_ across various pressure levels. At a temperature of 50 K, the EISF value for D_2_ reaches 1, corresponding to the narrow peak detected from QENS, implying complete confinement of D_2_ molecules within this material at this temperature. For both H_2_ and D_2_, the EISF profile retains a similar shape across all examined temperatures, pointing to a consistent type of molecular motion. Given the intricate dynamics of H_2_, further examination was pursued via HWHM analysis, which delineated two distinct phases of the H_2_ movement. The HWHM values from the broadenings of QENS peaks as a function of *Q* at all temperatures and pressures are plotted in Figure 7.

There are several established models to analyze QENS broadenings, each corresponding to different motion geometries of gas molecules, such as self-rotation, jump-diffusion, and vibration. Among the models used to fit the HWHM as a function of *Q*, the Singwi-Sjölander model^59^ (eq 5) stood out as the best fitting for the Lorentzian components of H_2_ across all temperatures and pressures (Figure 7a,b).

The model is traditionally used to illustrate the alternation between oscillatory and directed motion with the corresponding equation:
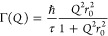
5where τ represents the time between two successive jumps in ps and *r*_0_ is the jumping length in Å. The self-diffusion coefficient *D*_s_ can be calculated with the mean-square-displacement ⟨*r*^2^⟩ = 6*r*_0_^2^:
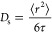
6

The Chudley–Elliot model,^48,60^ as described in eq 7, emerged as the optimal fit for the Gaussian peaks of H_2_, as showcased in Figure 6c,d. The Chudley–Elliot model elucidates translational jump diffusion within Bravais lattices, implying that the hydrogen molecules are jumping from positions outside the pore windows due to the quantum effect.
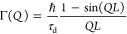
7where *L* is the mean jumping length and τ_d_ is the time needed for the guest to jump from one position to a new position. Correspondingly, the diffusion coefficient *D*_g_ can be calculated with . From both equations, at a small *Q* range, HWHM can be approximated to Γ(*Q*) = *DQ*^2^ (*Q* ≤ 0.2 Å^–1^) and *D* is the self-diffusion coefficient, while at high *Q* values, HWHM can be represented as .

This fitting performance aligns well with prior analyses that suggested that a fraction of the hydrogen molecules were not confined within the pores but instead moved freely around the material. The quantum effect probably acts as a barrier, limiting the ability of hydrogen molecules to access the pores. Consequently, the hydrogen molecules exhibited dual behaviors within the material: a confined motion in the pore window captured by the Lorentzian fitting and a jump-diffusion motion affected by the quantum effect represented by the Gaussian fitting of the QENS spectra.

In Figure 7e, the HWHM of the Lorentzian components for the QENS spectra of D_2_ at 77 K is presented. The HWHM value for confined D_2_ is notably lower than that of H_2_ at the same temperature, implying a more restrained or static motion for D_2_ in comparison to that for H_2_ at the same cage window. Additionally, the consistent HWHM of D_2_ across the measured *Q* range further substantiates this interpretation. The fact that it can be accurately represented by a horizontal line serves as evidence for the highly localized or immobile phase of D_2_ when confined within the pores.^50,61^

An increase in temperature results in higher HWHM values since the atoms move faster with the kinetic energy of the molecules increase. For the localized H_2_ (Lorentzian fitted, Figure 7a,b), the effect of temperature was pronounced compared to the jumping H_2_ molecules (Gaussian fitted, Figure 7c,d) meaning the loss in kinetic energy affected the adsorbed molecules more compared to the atoms already undergoing quantum effect. Moreover, the movement generated from the quantum effect was almost 10 times slower than the confined molecules in the pores, as suggested by HWHM, suggesting that the major movement in the porous system was contributed by the gas molecules in the confined pores.

On the contrary, the HWHM also decreased with pressure increase for both H_2_ and D_2_, which was attributed to the denser occupancy of the pores. As more guest molecules were packed into the available space with a pressure increase, the freedom of movement and the degree of jump-diffusion can be potentially reduced. Accordingly, the localized, confined motion becomes more dominant, reducing the observed broadening in the QENS spectra. Also, the influence of the adsorbent pressure could be more clearly detected at a lower temperature when the molecules were more easily detectable.

**Figure 8:**
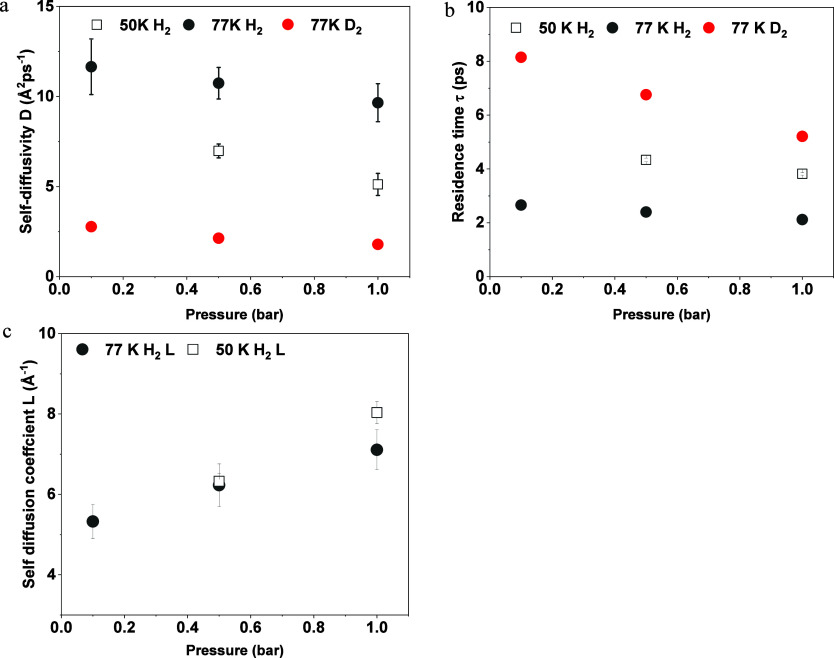
Self-diffusion coefficient (D_S_ Å^2^ps^–1^) and residence time (τ in ps) of D_2_ and H_2_: (a) D_S_ from Lorentzian fitted peaks of D_2_ (red) and H_2_ (black) at 50 (solid) and 77 K (open). (b) τ from Lorentzian fitted peaks of D_2_ (red) and H_2_ (black) at 50 (solid) and 77 K (open). (c) Gaussian diffusion length (*L*) in black of H_2_ at 50 (open square) and 77 K (solid circle).

More information is given by the fitted results of the self-diffusion coefficient (D_s_) and residence time (τ) of the isotopes (Figure 8, detailed numbers plotted in Tables S1 and S2). The self-diffusion coefficient represents the probability of the adsorbed isotopes successfully diffusing from one position to another position, and the residence time of the isotopes represents the time required for adsorbed molecules to jump from one pore to other pores.^62^

For both H_2_ and D_2_, the diffusivity of adsorbed gas molecules decreased with temperature, indicating the lower temperature helped the localization of the molecules. An increase in pressure (more gas molecules occupied the system) also led to a slightly lower diffusion coefficient as the availability of pores decreased (Figure 8a,b). Similarly, due to the limited kinetic energy at the lower temperature, the diffusivity of the molecules was restricted with lower diffusion coefficients and longer residence time. Unlike D_2_, whose residence time increased significantly (from localized to close to immobile) with a temperature decrease from 77 to 50 K, the residence time of confined H_2_ in the pore windows increased linearly from around 2.3 to 3.8 ps at 0.5 bar with decreasing temperature.

Compared to the confined H_2_ in the pores, where diffusivity was reduced from around 10 to around 7 Å^2^ ps^–1^ from 77 to 50 K at 0.5 bar, the heavier D_2_ molecules demonstrated a tendency to localize, exhibiting significantly slower diffusion speed. Specifically, D_2_’s diffusion was dropping to below 1 Å^2^ ps^–1^ at 50 K. That means the decrease in temperature led to a higher probability of finding the heavier D_2_ gas molecules in the restricted aligning with observations of increased EISF and corroborating previous studies.^54^ For the lighter H_2_ molecules, the residence time did not increase largely (2.4 ps at 77 K, 4.3 ps at 50 K) compared to D_2_, indicating quantum behavior that reduces the preference for localization. Also, the movement caused by the quantum effect seemed independent of the temperature decrease with almost the same diffusion length and residence time at different temperatures (Figure 8c, detailed in Table S2) further proving that the jump-diffusion of H_2_ is different from the transitional diffusion process.

By contrast, adsorbed D_2_ preferred not to diffuse among the pores at temperatures of 77 K with a self-diffusion coefficient 5 times lower than H_2_. At 77 K, the occupancy and localization of D_2_ molecules were further characterized by a longer residence time than in the case of H_2_. In other words, D_2_ prefers to be localized on the same position of the surface once it is adsorbed. Thus, when the mixture of gas molecules passes through the porous surface, D_2_ is more likely to remain in the pore, while H_2_ prefers to diffuse through the surface.

Compared to H_2_ whose residence time remains stable at around 2.4 ps in 0.1 bar −1.0 bar (77 K), the residence time of heavier D_2_ molecules was more pressure dependent at 77 K with the residence time decreasing from 16.2 to 10.4 ps, indicating the diffusion of D_2_ at 77 K is affected by the available porosities. Combining the information from both the self-diffusion coefficient and residence time, the kinetic difference between adsorbed H_2_ and D_2_ benefits from pressure decrease, suggesting that the separation of the isotopes can be enhanced at lower pressure.

On the contrary, the self-diffusion length (*L*, Figure 8c) for H_2_ molecules that experienced a quantum effect showed an opposite dependence on pressures: the increase in pressure increased the diffusion length. As more molecules were able to overcome quantum barriers, successful free jumping was more likely to happen. Compared to the self-diffusion coefficient generated from the molecules in the confined pores, the self-diffusion length collected from molecules undergoing quantum effects was more independent of the temperature decrease. In other words, the diffusivity of the H_2_ being adsorbed seemed more sensitive and could be controlled by temperature or pressure modification, while the diffusion of atoms undergoing quantum effect is more independent of the temperature change.

## Conclusions

In this study, we examined the dynamic behaviors of hydrogen isotopes, H_2_ and D_2_, within specific quantum porous systems (porous organic cages with a pore size of 3.6 Å) across varying temperatures and pressures using an array of experimental techniques. Notably, the experiments showcased quantifiable differences in the kinetic behavior of H_2_ and D_2_ within the porous system. Kinetic analysis of the sorption process highlighted that D_2_ adsorbed and equilibrated on the porous surface more rapidly than did H_2_. The Compton scattering results revealed the subtle interplay among the local binding, the potential energy landscape, and temperature influence on ZPE in H_2_ and D_2_ due to the isotope effect. The results suggested the potential of generating a quantum effect of H_2_ at 77 K and also confirmed that the maximum momentum difference was achieved at a liquid nitrogen temperature. Concurrently, QENS provided pivotal insights, accentuating the localized behavior of D_2_ and the augmented mobility of H_2_.

Based on the QENS results, the behavior of H_2_ within the porous system was characterized by two motion types. One illustrated a free rotational behavior within the cage window, as captured by the Lorentzian component of QENS spectra, while the other denoted a free jump-diffusion motion on the lattice surface, depicted by the Gaussian component. The confined H_2_ in the cage window showed a much higher probability of diffusion among the pores compared to D_2_ and the H_2_ underwent the quantum effect, further increasing the kinetic difference between adsorbed H_2_ and D_2_. The D_2_ isotope exhibited a pronounced localized behavior in the quantum system, which was made evident by the low self-diffusion coefficient, independent HWHM values of energy transfer (*Q*), and its notably high EISF value, especially compared with H_2_ at equivalent temperatures. This was further corroborated by the observation that the adsorbed D_2_ molecules exhibited a substantially reduced diffusion coefficient—approximately one-sixth of that of H_2_ at 0.1 bar—and a residence time that was nearly seven times longer.

Although the quantities of the adsorbed gas molecule varied primarily due to temperature differences, the kinetic differences and divergent motion modes between H_2_ and D_2_ could be pivotal in enhancing the separation performance, particularly at liquid nitrogen temperatures. Combining the information from the fractional update, NCS, and QENS results, it can be concluded that D_2_ is preferred by the POC and adsorbed on the available porous surface faster than H_2_. Once D_2_ is adsorbed to the POC system, it is much more localized and immobile than H_2_ with a low diffusion coefficient and long residence time due to the quantum effect of H_2_. The influence of the quantum barrier on the kinetic behavior of the two isotopes peaks at around 77 K, furnishing the optimal conditions for H isotope separation. Such findings suggest a promising potential for kinetic-based hydrogen isotope separation in the quantum system at 77 K. For example, using a breakthrough system^63^ for mixed H_2_ and D_2_ gas at 77 K, high purity H_2_ can be collected at the beginning and high purity D_2_ can be collected by degassing.

In conclusion, this comprehensive study underscores the profound impact of differences in the dynamics of isotopes on their behavior within quantum porous systems. Such insights are invaluable as they pave the way for designing efficient hydrogen isotope separation systems leveraging the unique principles of quantum sieving.

## Supporting Information Available

Synthesis of 6ET-RCC3, characterization of 6ET-RCC3, kinetic data and LDF fitting, neutron Compton scattering isotope spectra, QENS spectra and fitting, self-diffusivity, and residence time (PDF)

## ABBREVIATIONS

AT: acetone tied
BET: Brunauer–Emmett–Teller
CAQS: chemical affinity quantum sieving
CC3: covalent cage 3
CCS: carbon capture and sequestration
CD: cryogenic distillation
CE: Chudley–Elliot
EISF: elastic incoherent scattering factor
ET: ethanol tied
FD: Fickian diffusion
HWHM: half-width half maximum
INS: inelastic neutron scattering
KQS: kinetic quantum sieving
MOF: metal–organic framework
NCS: neutron Compton scattering
NMD: nuclear momentum distribution
PES: potential energy surface
PSD: pore size distribution
POC: porous organic cage
QB: quantum barrier
QENS: quasielastic neutron scattering
QS: quantum sieving
SS: Singwi–Sjölander
ZPE: zero-point energy
