# Supplementary material for: Investigation of the Dynamic Behaviour of H2 and D2 in a Kinetic Quantum Sieving System
Source: ACS Appl Mater Interfaces. 2024 Feb 29;16(10):12467–78. doi: 10.1021/acsami.3c17965 (PMC10941075; doi:10.1021/acsami.3c17965)
Supplement: Supplementary file 1 — am3c17965_si_001.pdf [file am3c17965_si_001.pdf]

Supporting information

Investigation of the dynamic behaviour of H_2 and D_2 in a kinetic quantum sieving system

Dankun Yang[a], Sebastien Rochat[b,c], Matthew Krzystyniak[d], Alexander Kulak[e], Jacques Olivier[f], Valeska P. Ting[a, g], Mi Tian[h*].

a. Department of Mechanical Engineering, University of Bristol, Bristol BS8 1TR, UK.
b. School of Engineering Mathematics and Technology, University of Bristol, Bristol BS8 1TW, UK.
c. School of Chemistry, University of Bristol, Bristol BS8 1TS, UK
d. ISIS Facility, Rutherford Appleton Laboratory, Didcot OX11 0QX, UK
e. School of Chemistry, University of Leeds, Leeds LS2 9JT, UK.
f. Institute Laue Langevin, France
g. School of Engineering, Computing and Cybernetics & Research School of Chemistry, Australian National University, Canberra 0200, Australia.
h. Department of Engineering, University of Exeter, EX4 4QF, UK
M.Tian@exeter.ac.uk

1. Synthesis of 6ET-RCC3

1. **Synthesis of cage 3-R**. Dichloromethane (100 ml) was layered slowly onto solid triformylbenzene (TFB, 5 g, 30.86 mmol) without stirring at room temperature. Trifluoroacetic acid (0.1 mL) was added directly to this solution as a catalyst for the imine bond formation. Later on, a solution of (R, R)-1,2- diaminocyclohexane (5 g, 44.64 mmol, 98%, Alfa Aesar) to form CC3-R cage in dichloromethanes (100 mL) was added, again without mixing. The reaction was covered and left to stand. The absence of stirring is important since it controls the dissolution rate of the TFB. Crystals could be observed on the liquid surface after around 30 minutes; to generate enough product, cages were collected after 5 days of reaction. The crystalline product was removed by filtration and washed with 95% ethanol / 5% dichloromethane solution, yielding around 5 g dried sample for the C3-R cage.

2. **Synthesis of RCC3**. The imine cage CC3-R (926 mg, 0.83 mmol) was dissolved in a $CHCl_3$ / methanol mixture (50 mL each) under stirring. Once the solution became clear, sodium borohydride ($NaBH_4$, 1.00 g, 26.5 mmol) was added, and the reaction was stirred for a further 12 hours at room temperature before water (2 mL) was added. The reaction was then stirred for a further 12 hours. The solvent was then removed under vacuum, resulting in a milky-like solid, which was extracted with chloroform (2 × 50 mL). The combined organic phase was washed with water (2 × 100 mL), and the $CHCl_3$ phase was dried using anhydrous $MgSO_4$ before being removed under vacuum, yielding around 92% crude RCC3.

3. **Purification of RCC3**. Taking advantage of the aminal reversibility of AT-RCC3 (AT stands for acetone) in solution, RCC3 crystals were dissolved in acetone (around 100 mg RCC3 in 10 -15 mL acetone). The solution was then covered and left to stand. Crystals started appearing on the wall and bottom of the vials shortly. The crystals (AT-RCC3) were collected after one day by filtration. AT-RCC3 was then dissolved in a $CHCl_3$ / CH_3OH mixture (1:1 v/v) by stirring. After 12 hours, several drops of distilled water (2 mL) were added to the solution, and the mixture was stirred for another 12 h. After removing the solvents, pure RCC3 was collected (approximate yield of 60%)

4. **Synthesis of 6ET-RCC3**. Acetaldehyde (200 mg, 4.55 mmol) was dissolved in MeOH (10 mL) and stirred at 0 ˚C. RCC3 (500 mg, 0.438 mmol) in MeOH (20 mL) was added to the solution. A white precipitate appeared upon the addition of RCC3. The reaction was stirred for a further 2 h at room temperature and collected by filtration. 6ET-RCC3 (around 75%) was obtained after washing the product with MeOH (3 x 10 mL) and drying the sample, giving 6ET-RCC3 an 82% yield.

2. Characterisation information

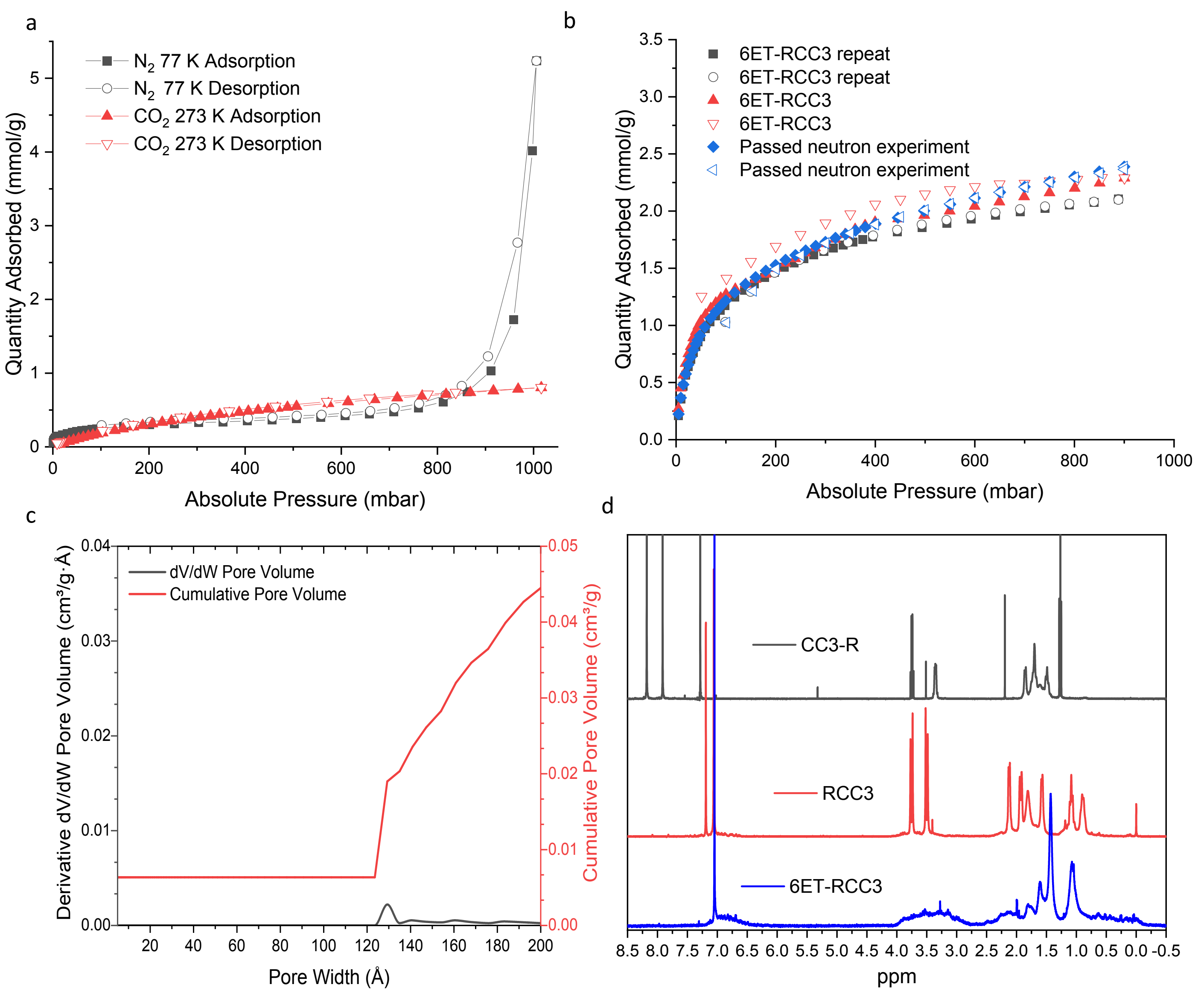

Figure S1: a) N_2 sorption at 77 K and CO_2 sorption at 273 K on 6ET-RCC3. b) H_2 sorption results at 77 K on 6ET-RCC3 just after synthesis and after neutron experiments. c) Pore surface distribution analysed with DFT performed on N_2 sorption isotherm measured at 77 K. d) ^{1}H-NMR spectra at room temperature for starting cage CC3, parent cage RCC3 and 6ET-RCC3 in $CDCl_3$.

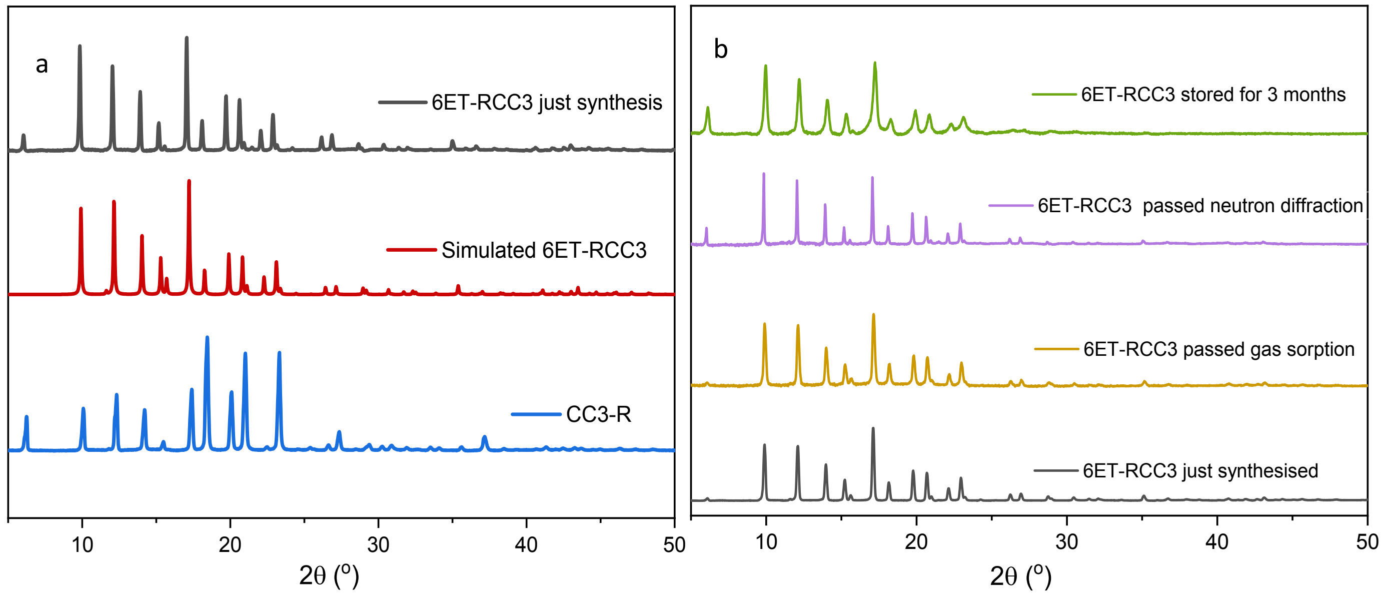

Figure S2: a) newly synthesised crystals; simulated 6ET-RCC3 PXRD pattern; parent cage CC3-R. b) PXRD pattern (from top to bottom) for sample stored for 3 months; after Compton experiments; after gas sorption; newly synthesised.

3. Kinetic information

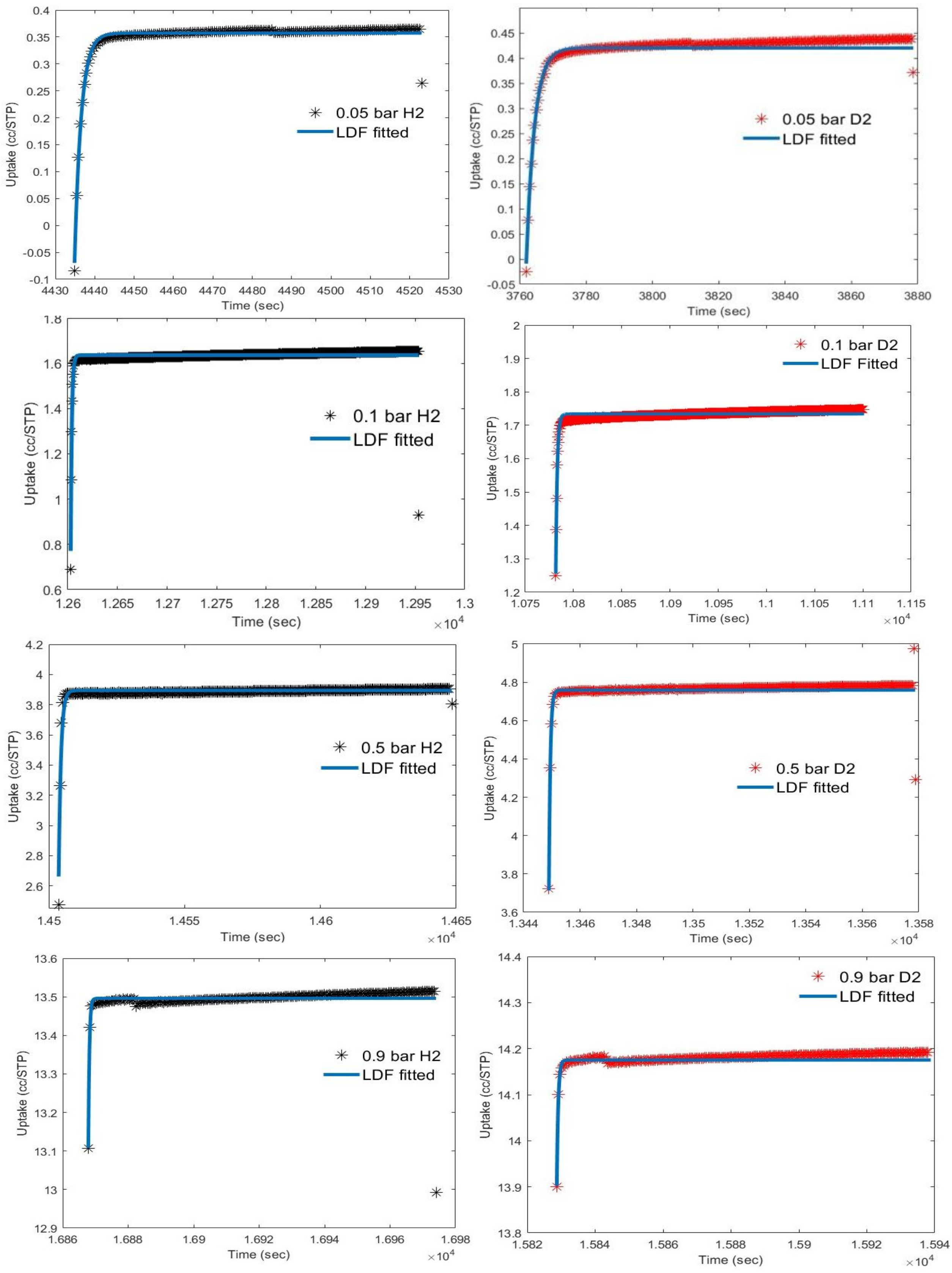

Figure S3: Examples of fitted fractional steps for H_2 (left) and D_2 (right) at 77 K with linear driving force model at 0.1 bar, 0.5 bar and 0.9 bar.

4. Neutron Compton scattering isotope-resolved spectra

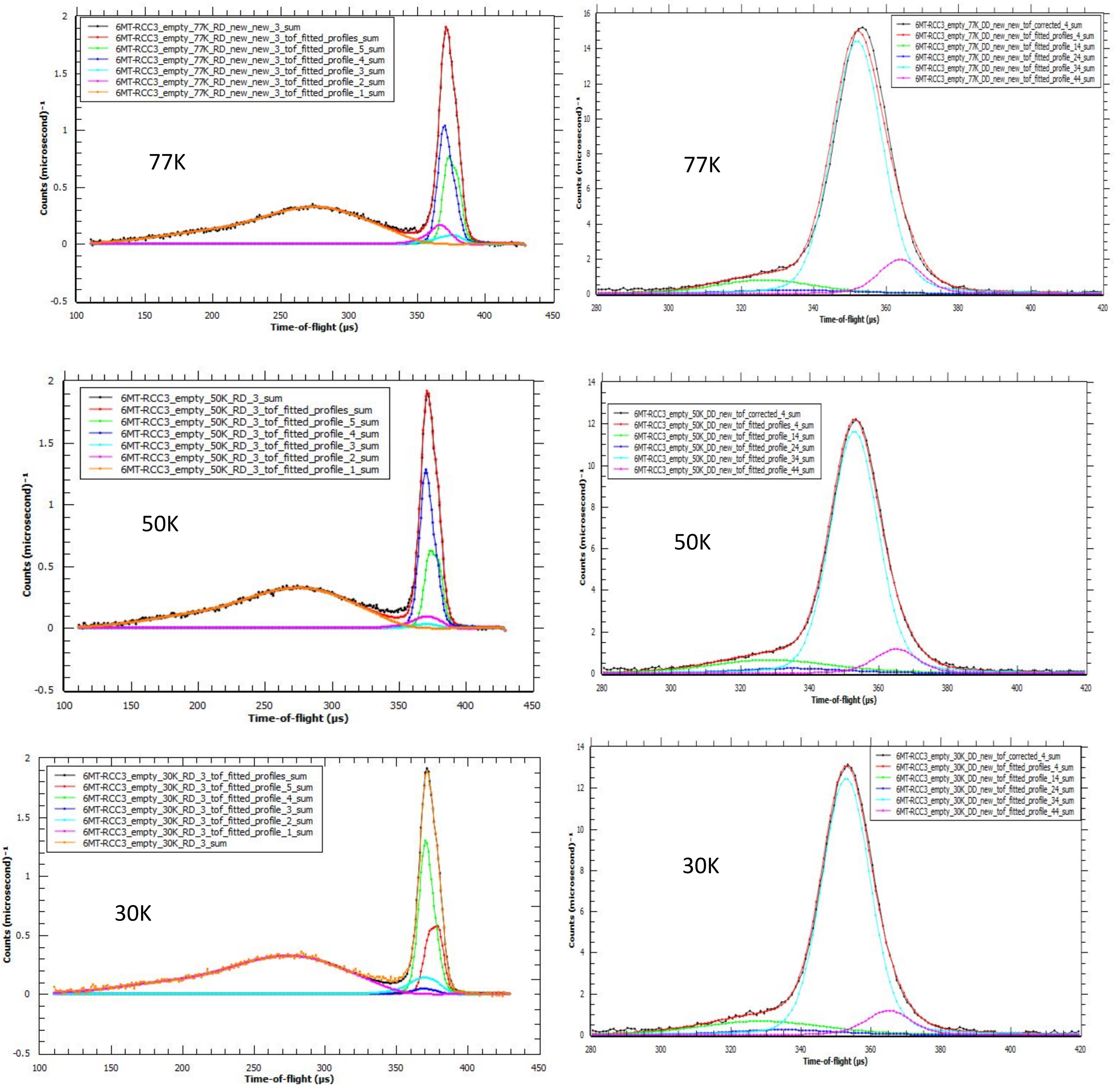

Figure S4: Forward Compton scattering results (left) for 6ET-RCC3 with H_2 loading: (black curve: corrected scattering output, green curve: H peak, deep blue curve: C peak, light blue: N peak, pink curve: Al peak (sample holder)); Back neutron scattering data (right) for 6ET-RCC3 before gas loading: (black curve: total fitting, green curve: fitting of C peak, deep blue: fitting of Al peak (sample holder), red curve: fitting of N peak).

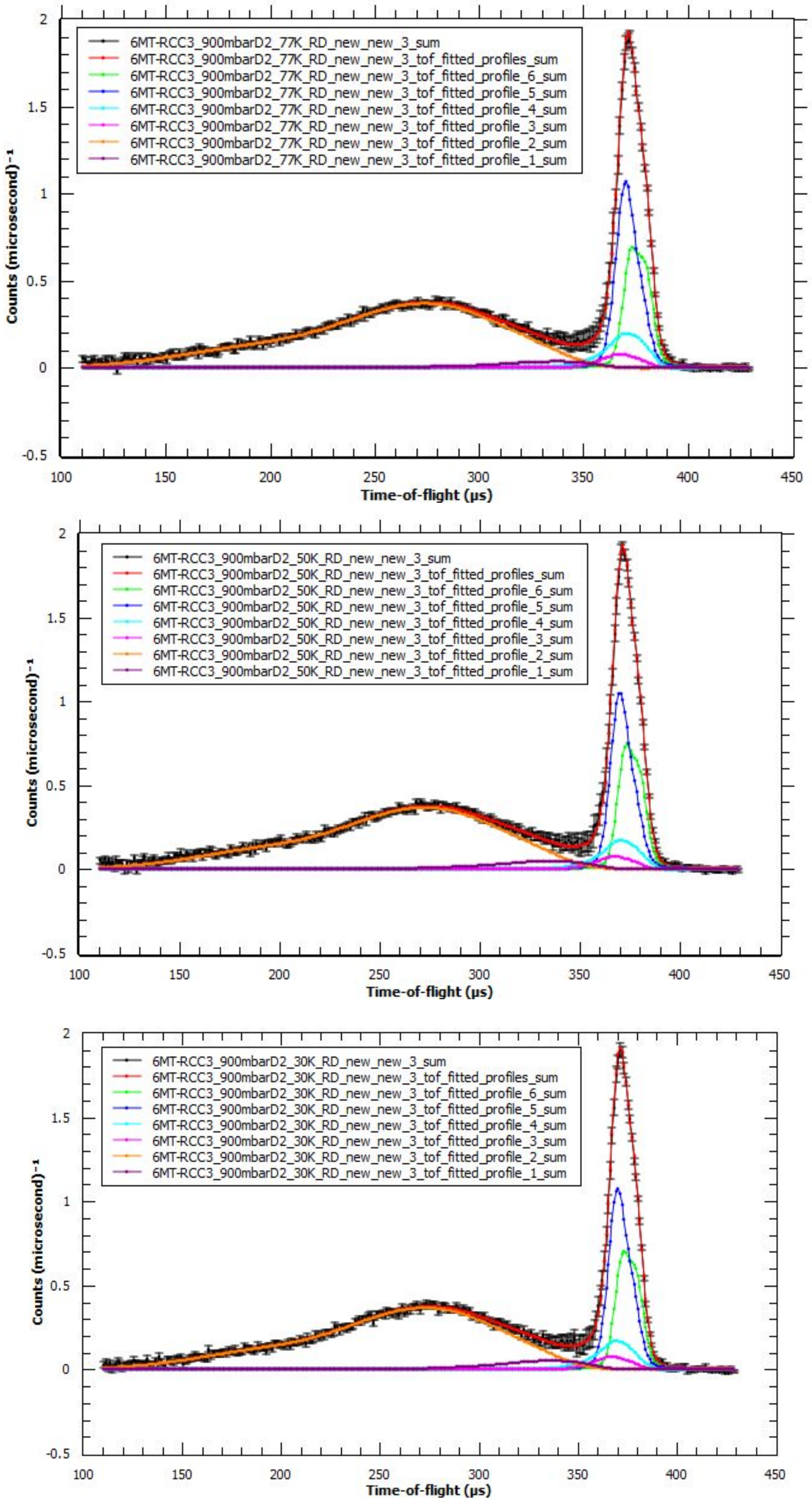

Figure S5: Forward Compton scattering for 6ET-RCC3 with D_2 loading: black curve: corrected scattering output; purple curve: D recoil peak; green curve: H peak, deep blue curve: C peak, light blue: N peak, pink curve: Al peak (sample holder).

5. QENS information

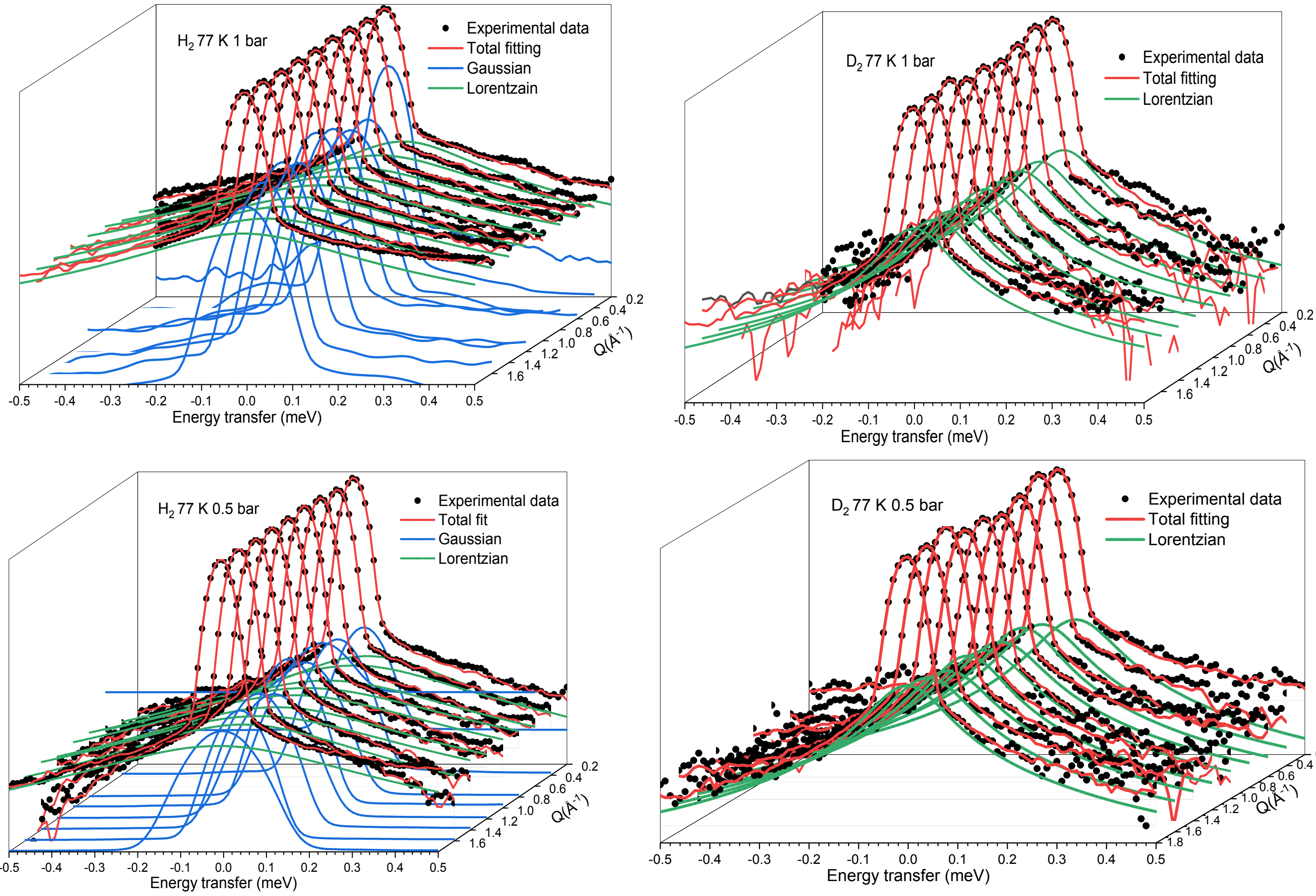

Figure S6: Log plotted fitted total QENS spectra for H_2 (left) and D_2 (right) at 1 bar (top) and 0.5 bar (bottom) 77 K (red for total fitting; blue for Gaussian fitting and green for Lorentzian fitting)

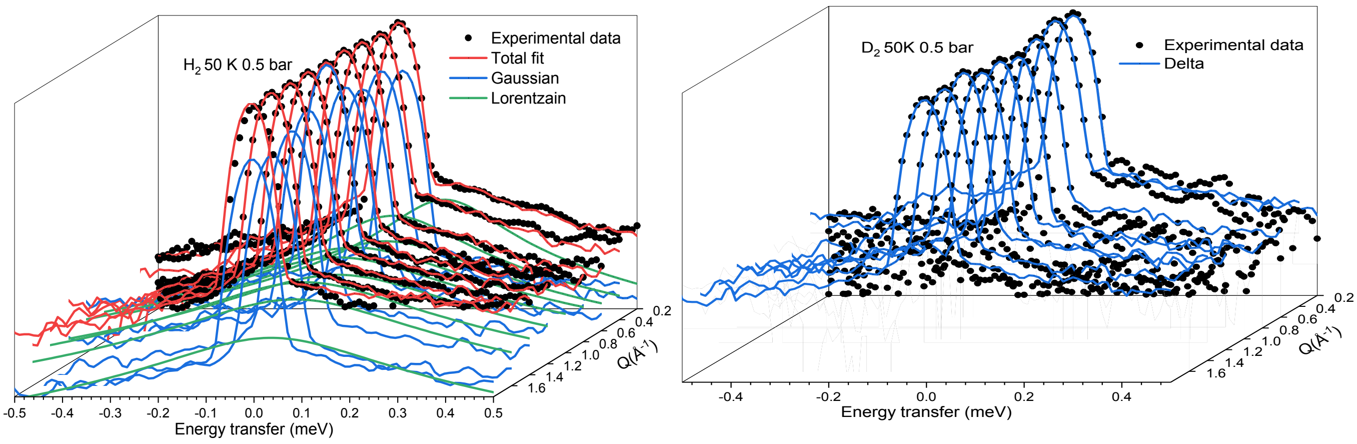

Figure S7: Log plotted fitted total QENS spectra for D_2 (right) and H_2 (left) at 0.5 bar 50 K (red for total fitting; blue for Gaussian fitting and green for Lorentzian fitting).

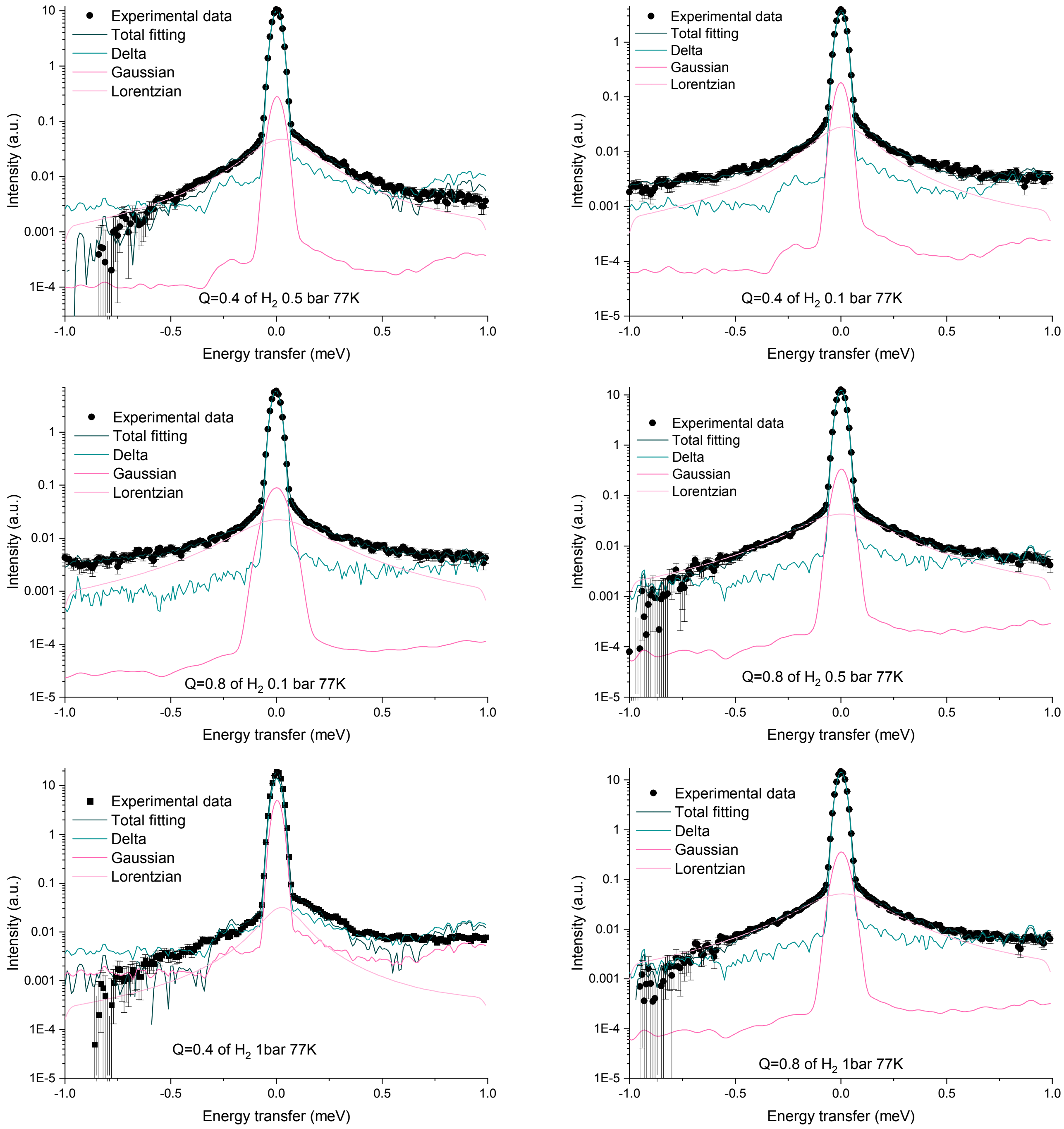

Figure S8: Fitted spectra for H_2 at 77 K at different Q levels and pressures (black for total fitting; green for delta; pink for Gaussian and light pink for Lorentzian.).

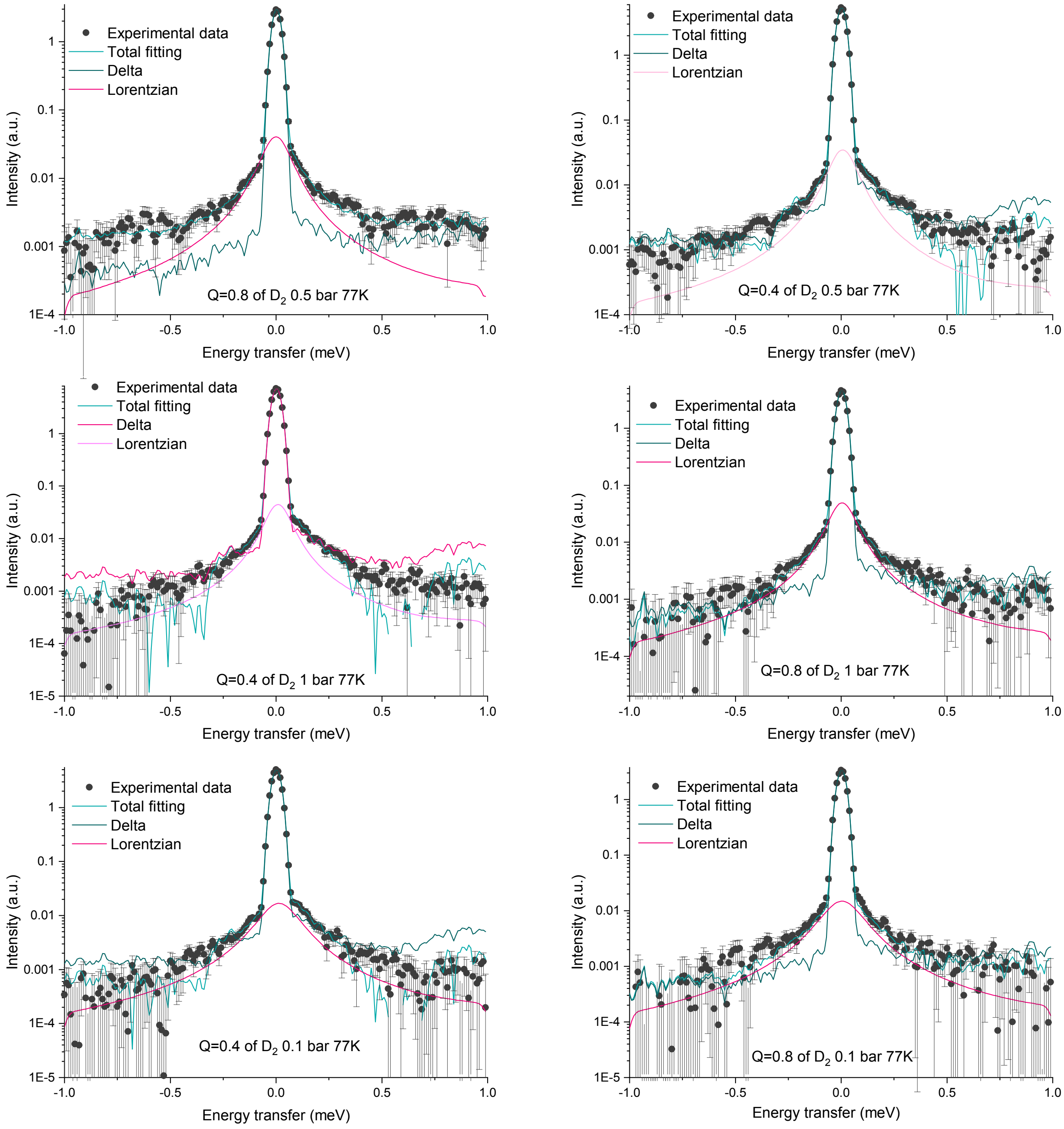

Figure S9: Fitted QENS spectra at 77 K for D_2 at different Q levels and different pressures (Black for total fitting; green for delta; pink for Lorentzian).

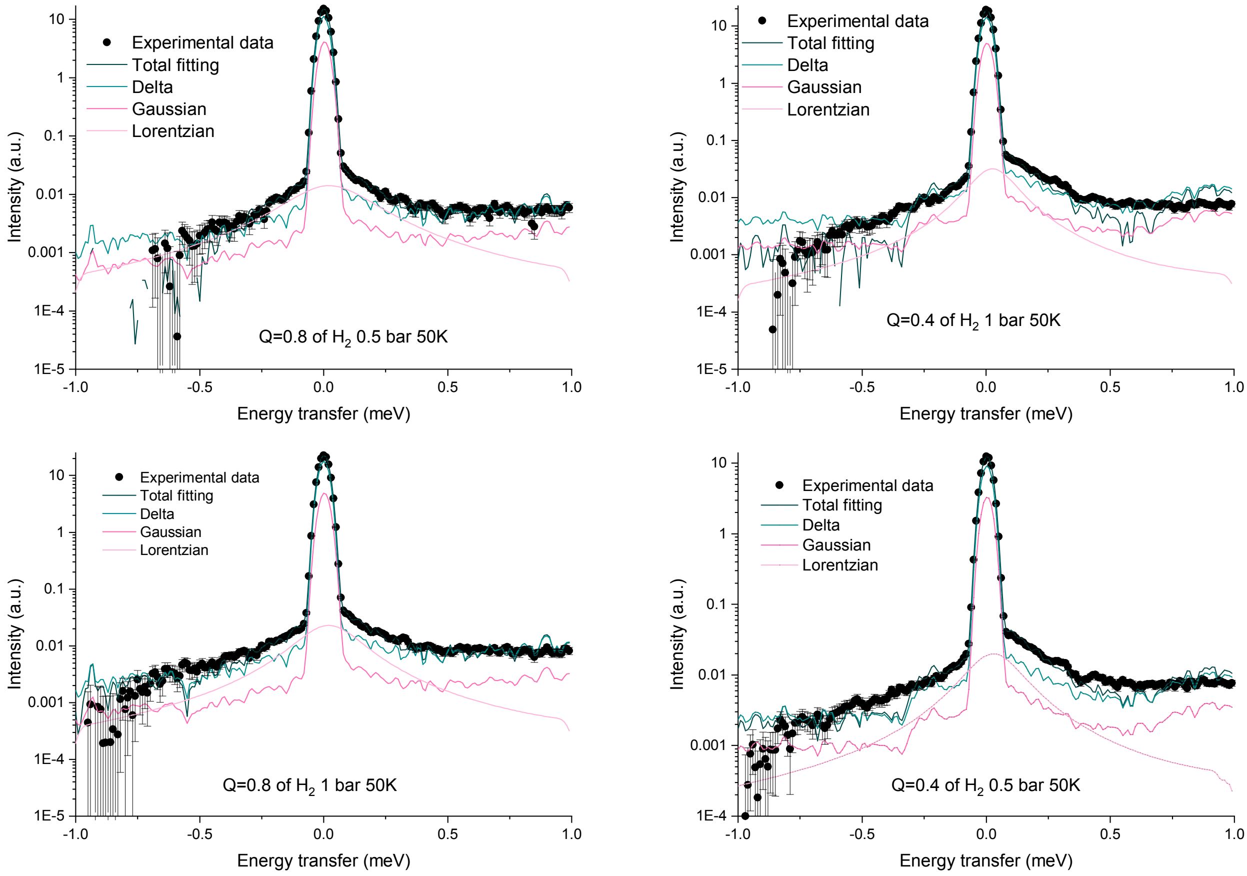

Figure S10: Fitted spectra at 50 K for H_2 with different Q levels and pressures (Black for total fitting; green for delta; pink for Gaussian and light pink for Lorentzian).

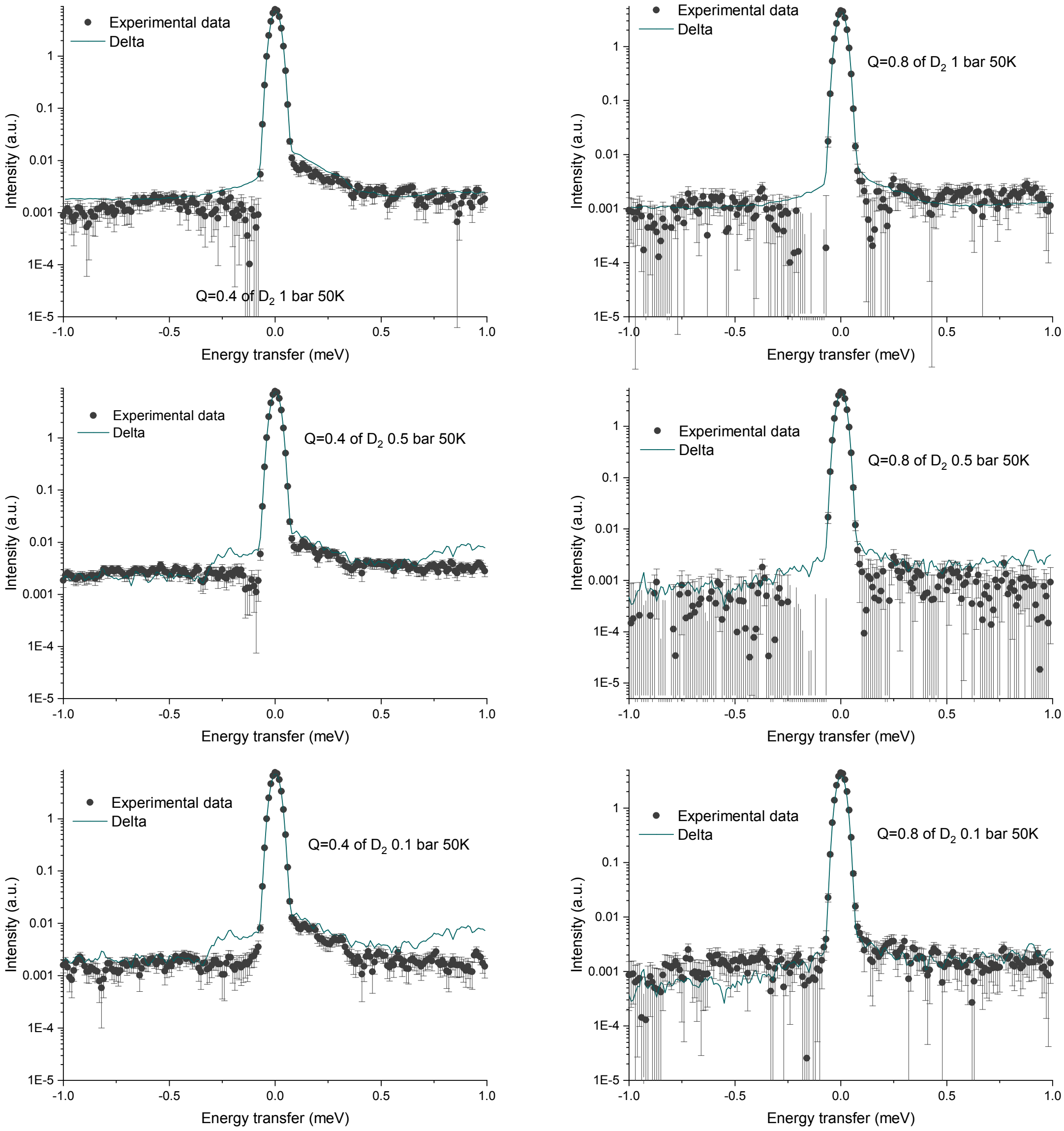

Figure S11: Fitted spectra for D_2 at 50 K with different Q levels and pressures (green for delta/ resolution).

Table S1: Self-diffusivity (D) and residence time (τ) of H_2 and D_2 at 50 K and 77 K

Pressure (bar)	D_H 77 K ($Å^2ps^{-1}$)	D_D 77 K ($Å^2ps^{-1}$)	D_H 50 K ($Å^2ps^{-1}$)	τ_H 77 K (ps)	τ_D 77 K (ps)	τ_H 50 K (ps)
**0.1**	11.65±1.54	2.40±0.08		2.66±0.06	16.29±0.28	
**0.5**	10.74±0.88	1.85±0.07	6.98±0.39	2.40±0.02	13.51±0.21	4.33±0.07
**1**	9.66±1.06	1.55±0.02	5.12±0.61	2.12±0.05	10.41±0.21	3.82±0.04

from Lorentzian peak fitting.

Table S2: Self-diffusion length (L) and residence time (τ_e) of H_2 underwent quantum effect at 50 K and 77 K from Gaussian peak fitting.

Pressure (bar)	L_H 77 K ($Å^{-1}$)	L_H 50 K ($Å^{-1}$)	τ_{eH} 77 K (ps)	τ_{eH} 50 K (ps)
**0.1**	5.32±0.42		10.65±0.74	
**0.5**	6.22±0.53	6.33±0.19	12.98±0.81	14.01±0.63
**1**	7.11±0.49	8.03±0.27	13.84±0.31	14.57±0.35
